# Comprehensive analysis of vulnerability status and associated affect factors among prehospital emergency patients: a single-center descriptive cross-sectional study

**DOI:** 10.3389/fpubh.2024.1330194

**Published:** 2024-02-29

**Authors:** Jiange Zhang, Ning Ding, Xue Cao, Shuting Zang, Ying Ren, Lijie Qin, Lijun Xu, Yanwei Cheng, Hongyan Li

**Affiliations:** ^1^Department of Emergency, Henan Provincial People’s Hospital, People’s Hospital of Zhengzhou University, People’s Hospital of Henan University, Zhengzhou, China; ^2^Henan Provincial Key Medicine Laboratory of Nursing, Henan Provincial People’s Hospital, Zhengzhou, China; ^3^Department of Rheumatology and Immunology, Henan Provincial People’s Hospital, People’s Hospital of Zhengzhou University, People’s Hospital of Henan University, Zhengzhou, China

**Keywords:** prehospital emergency care, patient vulnerability, SPECI scale, disease severity, interdisciplinary collaboration

## Abstract

**Background:**

Prehospital emergency care is a critical but often understudied aspect of healthcare. Patient vulnerability in this setting can significantly impact outcomes. The aim of this study was to investigate the vulnerability status and to determine associated affect factors among prehospital emergency patients in China.

**Methods:**

In this cross-sectional study conducted in China, from April 2023 to July 2023, we assessed the vulnerability of prehospital emergency patients using the Safety in Prehospital Emergency Care Index (SPECI) scale. We conducted a detailed questionnaire-based survey to gather demographic and disease-related information. We employed the SPECI scale, consisting of two subscales, to evaluate patient vulnerability. Statistical analyses, including t-tests, ANOVA, and multiple linear regression, were used to identify factors associated with vulnerability.

**Results:**

The study included a total of 973 prehospital emergency patients, with a response rate of 81.9%. These patients exhibited a low-to-moderate level of vulnerability, with an average SPECI score of 14.46 out of 40. Vulnerability was significantly associated with age (particularly those aged 60 and above), disease severity (severe conditions increased vulnerability), disease type (circulatory diseases correlated with higher vulnerability), alterations in consciousness, and chronic diseases. Unexpectedly, digestive system diseases were negatively correlated with vulnerability.

**Conclusion:**

Addressing patient vulnerability in prehospital care is essential. Tailored interventions, EMS provider training, and interdisciplinary collaboration can mitigate vulnerability, especially in older patients and those with severe conditions.

## Introduction

Emergency Medical Services (EMS) play a pivotal role as the first line of care for individuals facing acute health crises (1, 2). Despite their critical role, the prehospital stage has traditionally received less attention compared to in-hospital care, even though it can significantly impact patient outcomes (3). One crucial yet often overlooked aspect of this prehospital stage is the issue of patient vulnerability, which can exacerbate the challenges faced by EMS providers and profoundly affect the success of subsequent medical interventions (4, 5).

Vulnerability, reflecting susceptibility to physical and emotional harm, renders individuals defenseless and exposed to assault. In the unique context of prehospital emergencies, care diverges significantly from hospital settings, creating situations of heightened vulnerability for patients. This susceptibility is intricately linked to patient safety events, particularly in the challenging prehospital environment where patients are often unconscious, disoriented, or dealing with significant pain and impaired mobility. Within this realm, patient vulnerability transcends the severity of medical conditions, emphasizing the inherent incapacity of individuals to control life circumstances or shield themselves from risks. This intricate phenomenon intertwines psychosocial and environmental determinants, incorporating factors like age, socioeconomic status, and comorbidities (6–8). These factors exert significant influence not only on immediate outcomes, such as survival and stabilization but also on long-term consequences, including quality of life and healthcare costs. Studies have consistently demonstrated that vulnerable patients often experience delays in receiving essential care due to various impediments, such as a lack of social support or transportation barriers (9–11). These delays can lead to adverse outcomes and an increased mortality rate among this vulnerable demographic (12).

Despite the ample evidence emphasizing the significance of addressing patient vulnerability, the prehospital phase remains a relatively uncharted territory in this regard, especially in China. This research gap becomes all the more concerning when considering the global aging population and the rising demands on healthcare services (13). Recognizing and addressing patient vulnerability in prehospital care is not just a matter of ethics but also strategic importance, as it can reduce healthcare costs, prevent complications, and enhance overall community health (14).

Hence, this study aims to address this gap in the literature by conducting a comprehensive analysis of vulnerability and its associated factors among prehospital emergency patients. The goal is to generate insights that could drive more personalized, effective, and equitable EMS interventions. The findings from this research can be of significant benefit to policymakers, healthcare administrators, and frontline EMS providers, potentially improving patient outcomes and optimizing resource allocation.

## Methods

### Study design, setting, and participants

This descriptive cross-sectional study was conducted at a Grade III A hospital in Zhengzhou, Henan Province, China, during the period from 1 April 2023 to 1 July 2023. The study focused on prehospital emergency patients. The inclusion criteria for participants were age 18 years or older and a willingness to participate. Patients who died before or during medical assistance provision, or those who declined to participate or could not provide informed consent, were excluded. The study adhered to the Strengthening the Reporting of Observational Studies in Epidemiology (STROBE) guidelines and obtained approval from the Ethics Committee of Henan Provincial People’s Hospital.

### Questionnaire

The questionnaire used comprises two sections: one for demographic and disease-related details (refer to Table 1 for all items), and the other for vulnerability assessment. Disease-related information is obtained by EMS providers through patient or witnesses interviews, on-site examinations (temperature, pulse, respiration, blood pressure), and necessary tests (e.g., rapid blood glucose, electrocardiogram). The prehospital emergency physician integrates this information to report the preliminary diagnosis and grade the severity based on experience and treatment impact.

**Table 1:** Demographic and disease-related characteristics of patients in prehospital emergency care (*n* = 973).

Variables	*n*	%
**Age**		
18~44 years	266	27.34
45~59 years	189	19.42
≥60 years	518	53.24
**Gender**		
Male	521	53.55
Female	452	46.45
**Alterations in the level of consciousness**		
Yes	102	10.48
No	871	89.52
**Receiving medical assistance**		
Yes	916	94.14
No	57	5.86
**Having chronic disease**		
Yes	456	46.87
No	517	53.13
**Disease type**		
Injury, poisoning and certain other consequences of external causes	288	29.60
Diseases of the nervous system	227	23.33
Diseases of the respiratory system	162	16.65
Diseases of the circulatory system	111	11.41
Diseases of the digestive system	74	7.60
Other	111	11.41
**Disease severity**		
Mild	351	36.07
Moderate	537	55.19
Severe	85	8.74
**Location**		
Home	500	51.39
Traffic route	194	19.94
Medical institution	129	13.26
Public place	83	8.53
Workplace	25	2.57
Other	42	4.31
**Onset time (according to Beijing time)**		
00:00~07:59	213	21.89
08:00~15:59	409	42.03
16:00~23:59	351	36.04

For the assessment of vulnerability, we employed the Safety in Prehospital Emergency Care Index (SPECI) scale, originally developed by Antonio Montero García (4). The SPECI questionnaire assesses vulnerability in adult prehospital emergency patients, considering RESPIRATORY, MOBILITY, and SAFETY dimensions. Each dimension incorporates Condition Characteristics and Medical Interventions. Each item of the SPECI scale, or each question, sets 5 evaluation criteria, assigning 1, 2, 3, 4, and 5 points, respectively, based on corresponding criteria (refer to Supplementary material 1 for detailed scoring rules). The 5-point Likert scales evaluate factors like respiratory rate, mobility, and awareness. Scores range from 8 to 40, with lower scores indicating lower vulnerability. This tool aids healthcare professionals in comprehensive vulnerability evaluation post-initial assistance, providing insights into patient safety.

To ensure cultural appropriateness and linguistic accuracy, the researchers contacted the original scale’s authors and obtained permission to translate it into Chinese. The translation process included forward and backward translation, comparison with the original scale, and cross-cultural adaptation via psychometric techniques. The Chinese version of the SPECI scale exhibited commendable reliability (McDonald’sω = 0.900; Composite Reliability, CR = 0.848; Average Variance Extracted, AVE = 0.574). In terms of construct validity, exploratory and confirmatory factor analyses were conducted with a substantial sample size of 480 subjects. The two-factor structure explained 76.857% of the total variance. Confirmatory factor analysis demonstrated favorable fit indices, including minimum discrepancy per degree of freedom (χ2/df) of 3.375, Comparative Fit Index (CFI) of 0.967, Incremental Fit Index (IFI) of 0.967, Normed Fit Index (NFI) of 0.954, and Root Mean Square Error of Approximation (RMSEA) of 0.099. These detailed analyses affirm the robustness and accuracy of the Chinese SPECI scale in assessing vulnerability among prehospital emergency patients.

### Data collection

All surveys were conducted by trained medical investigators. Patients were initially briefed on the research’s content and significance. Informed consent was obtained from each patient before administering the paper questionnaire. For patients unable to independently complete the questionnaire, investigators objectively recorded the patient’s responses and cross-verified them after ensuring that the patient’s views were fully understood. Upon completion of the questionnaire, an on-site review was conducted, and any missing or incomplete information was promptly addressed.

### Data analysis

Descriptive statistics were employed to summarize demographic and disease-related characteristics within the study population. Continuous data were presented as mean ± standard deviation (M ± SD), while categorical data were expressed as absolute numbers and percentages. The Kolmogorov–Smirnov test was used to assess normality distributions of continuous variables. Normally distributed continuous variables were compared by Student t-test, while the continued variables that were not normally distributed were compared by Mann–Whitney *U* test. Categorical data were analyzed with Chi-square tests. Independent sample t-tests and one-way analysis of variance (ANOVA) were performed to identify differences and associations between demographic and disease-related variables and vulnerability. Variables with *p* < 0.10 in these initial analyses were included in subsequent multivariate analysis. To identify the most significant variables associated with vulnerability among demographic and disease-related factors, a multiple linear regression model was employed. All *p*-values were two-sided, and *p* < 0.05 was considered statistically significant. The data analysis was conducted using SPSS statistical software version 25.0 (IBM Corporation, Armonk, NY).

## Results

### Demographic and disease-related characteristics of patients in prehospital emergency care

Out of the 1,210 distributed questionnaires, 991 were returned, indicating an 81.9% response rate. Among these, 18 were excluded as they pertained to individuals who died either before or during the provision of medical assistance. Consequently, a total of 973 questionnaires were subjected to analysis in this study.

Table 1 summarizes the demographic and disease-related characteristics of patients receiving prehospital emergency care. Notably, the majority of patients were aged 60 or above (53.24%), with 53.55% being male. A notable proportion, 10.48% of patients, experienced alterations in consciousness, while the vast majority, 94.14%, received medical assistance. Furthermore, nearly half of the patients reported the presence of chronic diseases (46.87%). The most prevalent disease category observed was ‘Injury, poisoning, and certain other consequences of external causes’ (29.60%). Disease severity exhibited a spectrum ranging from mild (36.07%) to moderate (55.19%) and severe (8.74%). Incidents occurred most frequently at patients’ homes (51.39%), with daytime hours, particularly 08:00~15:59, being the most common onset time (42.03%).

### Vulnerability of patients in prehospital emergency care

The vulnerability of patients who received prehospital emergency care was assessed using the SPECI scale, and the results are summarized in Table 2. The total scale score, spanning from 8 to 40, exhibited a mean score of 14.46 (SD = 6.01). In terms of the “Respiratory and Medical Interventions Safety” dimension, scores ranged from 3 to 15, with a mean score of 4.12 (SD = 2.14). Conversely, the “Condition Characteristics Safety and Mobility” dimension had scores ranging from 5 to 25, with a mean score of 10.35 (SD = 4.64). It is noteworthy that for all measures, the skewness and kurtosis values were 0.078 and 0.157, respectively.

**Table 2:** The SPECI scores of prehospital emergency patients (*n* = 973).

	Total score range	Total score	Skewness	Kurtosis
		Mean (SD)		
Total scale	8~40	14.46 (6.01)	0.078	0.157
Respiratory and medical interventions safety	3~15	4.12 (2.14)	0.078	0.157
Condition characteristics safety and mobility	5~25	10.35 (4.64)	0.078	0.157

### Univariate analysis of factors affecting vulnerability

Results from Table 3 reveal the factors affecting vulnerability among patients in prehospital emergency care. Patients aged 60 and above exhibited higher vulnerability scores, with males scoring higher than females. Alterations in consciousness, receipt of medical assistance, and the presence of chronic diseases were associated with elevated vulnerability scores. Disease type demonstrated variability, with circulatory system diseases linked to higher vulnerability scores, while digestive system diseases showed lower scores. Patients with severe conditions scored significantly higher on vulnerability. The incident location also played a role, with incidents in medical institutions correlating with higher scores. However, onset time, as per Beijing time, did not exhibit a significant correlation with vulnerability.

**Table 3:** Univariate analysis of factors affecting vulnerability (*n* = 973).

Variables	Score for the SPECI	t/F	*p*-value
	Mean (SD)		
Age		18.772^*^	0.000
18~44 years	12.76 (4.42)		
45~59 years	14.14 (6.62)		
≥60 years	15.46 (6.29)		
Gender		3.046^**^	0.002
Male	15.00 (6.55)		
Female	13.85 (5.27)		
Alterations in the level of consciousness		20.820^**^	0.000
Yes	28.27 (7.41)		
No	12.85 (3.01)		
Receiving medical assistance		3.724^**^	0.000
Yes	14.56 (6.14)		
No	12.84 (3.14)		
Having chronic disease		7.912^**^	0.000
Yes	16.08 (7.18)		
No	13.03 (4.28)		
Disease type		8.672^*^	0.000
Injury, poisoning and certain other consequences of external causes	13.09 (4.09)		
Diseases of the nervous system	15.15 (5.67)		
Diseases of the respiratory system	15.67 (6.58)		
Diseases of the circulatory system	16.29 (9.09)		
Diseases of the digestive system	12.64 (4.83)		
Other	14.27 (5.90)		
Disease severity		523.804^*^	0.000
Mild	12.19 (2.79)		
Moderate	13.77 (3.89)		
Severe	28.26 (8.49)		
Location		7.656^*^	0.000
Home	14.92 (6.18)		
Medical institution	16.24 (7.42)		
Workplace	13.00 (3.35)		
Public place	14.48 (6.26)		
Traffic route	12.78 (4.37)		
Other	12.17 (3.60)		
Onset time (according to Beijing time)		0.374^*^	0.675
00:00~07:59	14.67 (6.35)		
08:00~15:59	14.27 (5.39)		
16:00~23:59	14.56 (6.48)		

^*^F-value, ^**^t-value.

### Multiple linear regression analysis of factors affecting vulnerability

In the multiple linear regression analysis, eight variables with *p* < 0.10 from the univariate analysis were included as predictors in a stepwise model with the SPECI score as the dependent variable. The final model exhibited an *R*^2^ value of 0.741 and an adjusted *R*^2^ value of 0.736 (*F* = 151.663, *p* < 0.001), signifying that 73.6% of the variation in SPECI scores could be explained. The analysis revealed that being aged 60 and above (B = 8.180, t = 16.458, *p* = 0.000), having a moderate disease severity (B = 0.752, t = 3.201, *p* = 0.001), and severe disease (B = −8.618, t = −2.086, *p* = 0.038), experiencing alterations in consciousness (B = 10.930, t = 26.971, *p* = 0.000), and having chronic disease (B = 0.905, t = 3.784, *p* = 0.000) were positively correlated with vulnerability. Conversely, diseases of the digestive system were negatively correlated with vulnerability (B = −1.340, t = −2.845, *p* = 0.005; Table 4).

**Table 4:** Multiple linear regression analysis of factors associated with prehospital emergency patients’ SPECI scores (*n* = 973).

Model		B	SE	Beta	t	*p*-value
(constant)		11.565	0.694	-	16.665	0.000
Age (reference = 18~44 years)	≥60 years	0.660	0.287	0.055	2.300	0.022
Disease severity (reference = Mild)	Moderate	0.752	0.235	0.062	3.201	0.001
	Severe	8.180	0.497	0.384	16.458	0.000
Disease type (reference = Other)	Diseases of the digestive system	−1.340	0.471	−0.059	−2.845	0.005
Alterations in the level of consciousness (reference = No)	Yes	10.930	0.405	0.557	26.971	0.000
Having chronic disease (reference = No)	Yes	0.905	0.239	0.075	3.784	0.000

*R^2^* = 0.741; Adjusted *R^2^* = 0.736; F = 151.663; *p* < 0.001.

## Discussion

The present study sheds light on the vulnerability status of prehospital emergency patients, revealing a low-to-moderate level of vulnerability with an average SPECI score of 14.46 out of a possible 40. Key factors influencing vulnerability include age, disease severity, type, alterations in consciousness, and the presence of chronic diseases. These findings underscore the importance of tailored prehospital care strategies, specialized training for EMS providers, and interdisciplinary collaboration to address the unique needs of vulnerable patients. As prehospital emergency care plays a crucial role in patient outcomes, understanding and mitigating vulnerability factors are essential steps toward enhancing the quality and effectiveness of emergency medical services.

The study identified age as a significant predictor of vulnerability among prehospital emergency patients. Specifically, patients aged 60 and above exhibited higher vulnerability scores. This result aligns with the well-documented vulnerability of the older adult population, who often experience complex health issues, reduced physiological reserves, and psychosocial challenges (5, 15, 16). In the context of prehospital emergency care, older adults may face difficulties in accessing timely care due to mobility issues or a lack of social support (17, 18). Healthcare systems should consider tailored interventions for this demographic, such as geriatric-focused EMS training and specialized protocols for assessing and addressing the unique needs of older patients (19–22).

Likewise, the present study also showed that disease severity and type significantly impact patient vulnerability. Patients with severe conditions scored higher on vulnerability, emphasizing the critical role of disease acuity in prehospital care (4). It is essential for EMS providers to accurately assess the severity of a patient’s condition to allocate appropriate resources and interventions (23). Moreover, the association between disease type and vulnerability underscores the need for disease-specific protocols and training for EMS teams. For example, circulatory system diseases were linked to higher vulnerability scores, indicating the importance of rapid intervention in cases of cardiovascular emergencies (23).

Moreover, the study found that alterations in consciousness and the presence of chronic diseases were positively correlated with vulnerability. Impaired consciousness can complicate communication and assessment, making it essential for EMS providers to develop strategies for effectively evaluating and managing such patients (4, 24)^.^ Additionally, the presence of chronic diseases highlights the need for comprehensive care coordination between prehospital and in-hospital settings to ensure that patients receive appropriate follow-up care and medication management (25–28).

Interestingly, the study identified digestive system diseases as negatively correlated with vulnerability. This unexpected finding warrants further investigation and may suggest that patients with digestive system diseases are better prepared or equipped to manage emergencies (29, 30). Future research could explore the reasons behind this association and whether it holds true in different healthcare contexts.

In China, prehospital care providers undergo comprehensive training in various aspects of emergency medical services, covering theoretical knowledge and practical skills. However, there is currently no standardized training module specifically addressing patient vulnerability. Through the application of the SPECI scale, our study contributes valuable insights into the vulnerability assessment of prehospital emergency patients, offering a foundation for future considerations in the training and practice of prehospital care providers. Despite the significance of our findings, several limitations should be acknowledged when interpreting the results. Firstly, the single-center design may limit generalizability. To address this, future research could involve multiple healthcare settings and regions. Secondly, the response rate of 81.9% and the exclusion of patients who declined or could not provide consent may introduce sampling bias. Exploring alternative consent methods for incapacitated patients could mitigate this. Thirdly, while efforts were made to adapt the SPECI scale linguistically and culturally, nuances may remain unaddressed, suggesting a need for ongoing collaboration and validation. Additionally, the cross-sectional design provides a snapshot of vulnerability, and longitudinal studies could offer deeper insights. Moreover, reliance on self-reporting may introduce recall bias, emphasizing the importance of incorporating objective data sources. Furthermore, the study did not explore external factors like socioeconomic status and access barriers, which future research should consider. Despite these limitations, this study provides crucial insights into patient vulnerability in prehospital emergency care, and addressing these limitations in future research will contribute to a more comprehensive understanding of this critical healthcare issue.

## Conclusion

To the best of our knowledge, this study is the first investigation into the levels of vulnerability among prehospital emergency patients in China. The findings reveal that vulnerability levels among these patients were low-to-moderate. Notably, the study identified five key factors influencing vulnerability. Recognizing and addressing these factors can enhance the overall quality and effectiveness of prehospital emergency care, ultimately leading to improved patient outcomes and advancements in the field of emergency medical services.

## Data availability statement

The original contributions presented in the study are included in the article/Supplementary material, further inquiries can be directed to the corresponding authors.

## Ethics statement

The Ethics Committee of Henan Provincial People’s Hospital approved the study. The studies were conducted in accordance with the local legislation and institutional requirements. Written informed consent for participation was not required from the participants or the participants’ legal guardians/next of kin in accordance with the national legislation and institutional requirements.

## Author contributions

JZ: Data curation, Investigation, Methodology, Software, Writing – original draft. ND: Data curation, Software, Writing – review & editing. XC: Data curation, Investigation, Methodology, Software, Writing – original draft. SZ: Data curation, Investigation, Methodology, Software, Writing – review & editing. YR: Project administration, Supervision, Writing – review & editing. LQ: Project administration, Supervision, Writing – review & editing. LX: Project administration, Supervision, Writing – review & editing. YC: Project administration, Supervision, Writing – review & editing. HL: Writing – review & editing.
