# Supplementary material for: Comprehensive analysis of vulnerability status and associated affect factors among prehospital emergency patients: a single-center descriptive cross-sectional study
Source: Front Public Health. 2024 Feb 29;12:1330194. doi: 10.3389/fpubh.2024.1330194 (PMC10937386; doi:10.3389/fpubh.2024.1330194)
Supplement: Supplementary file 1 [file Data_Sheet_1.pdf]

SPECI: SAFETY IN PREHOSPITAL EMERGENCY CARE INDEX

INSTRUCTIONS FOR USE

This is an instrument designed to evaluate the vulnerability of adult patients (over 18 years) assisted in prehospital emergency settings, both conscious and unconscious. The scale can be complete by physicians, nurses or paramedic staff who work in the prehospital setting.

It consists of three dimensions: *RESPIRATORY, MOBILITY* and *SAFETY.*

Within each of these dimensions there are different types of factors: CONDITION CHARACTERISTICS (inherent to the clinical condition) or MEDICAL INTERVENTIONS (interventions carried out by the healthcare team). The final score is the sum of each section: Condition Characteristics Respiratory + Medical Interventions Respiratory + Condition Characteristics Mobility + Condition Characteristics Safety + Medical Interventions Safety.

The Condition Characteristics Safety block is composed, in turn, by several sections that are scored independently: consciousness / cognition, patient communication, risk factors, threats and patient coping with their health situation, and ability to protect against external threats.

The score ranges from 8 to 40. The lower the score, the lower the level of vulnerability and vice versa.

The instrument should be used after the initial assistance is finished, regardless of whether the patient is going to be transferred to a health centre, or not.

SCORING

A.- RESPIRATORY

CONDITION CHARACTERISTICS

Conscious and unconscious patients, with spontaneous ventilation and permeabilized airway are scored based on their respiratory rate:

- Greater than or equal to 11 breaths per minute (bpm) and less than or equal to 20, score 1.
- Greater than or equal to 21 bpm and less than or equal to 24, score 2.
- Greater than or equal to 25 bpm and less than or equal to 27, score 3.
- Greater than or equal to 28 bpm and less than or equal to 30, score 4.
- Patients with respiratory rates greater than 31 bpm or less than 11, or who have apnoea pauses, score 5.

Unconscious patients under mechanical ventilation score 4, if they are well adapted to the ventilatory pattern prescribed. Patients who are not well adapted to the ventilatory pattern score 5.

MEDICAL INTERVENTIONS

The Medical Interventions Respiratory block is evaluated accordingly with the devices used during the episode:

• The absence of devices score 1.

• The placement of a Venturi-type mask with FiO_2 up to 50%, or a nasal cannula score 2.

• Use of aerosol therapy masks, score 3.

• Oxygen administration by mask with reservoir bag, or non-invasive mechanical ventilation devices such as CPAP score 4.

• The need for isolation or permeabilization of the airway by any device (oropharyngeal or nasopharyngeal cannulas, supraglottic devices, endotracheal tube, surgical airways), score 5.

B.- MOBILITY

CONDITION CHARACTERISTICS

The score is calculated based on the ability of the patient to walk and perform movements in an autonomous way:

• Patients who walk normally, score 1.

• Patients who require vigilance, but not help while walking, or do so using any type of device (cane, walker, crutch ...), score 2.

• Patients who do not walk autonomously (although they can do so with help from another person), but can stand without any help and supporting themselves, score 3.

• Patients who do not walk, even with the help of another person, who cannot stand alone, but can sit without help, maintain body posture and move voluntarily and in a controlled manner, score 4.

• Patients who do not walk, or cannot stand, or do not maintain body posture or move uncontrolled in a sitting position or lack the ability to perform any movement autonomously, score 5.

C.- SECURITY

CONDITION CHARACTERISTICS

This dimension has several elements that score independently: awareness and cognition, ability to communicate, risk factors and coping style, and the ability to protect against external threats.

a.-Consciousness/cognition

• Patients who understand the information, obey orders, and have a score on the Glasgow Coma Scale (GCS) of 15, score 1.

• Patients who understand the information, obey orders, although they show a trend towards drowsiness (they easily awaken with mild stimuli, such as voice or touch), score 2.

• Patients who have a limited understanding of the information but obey orders, score 3. They could have GCS 15, or show confusion. Examples: post-critical patients, with mild cognitive impairment, with pain or dyspnoea, or after a traumatic brain injury who, in any case and despite their condition, obey orders.

• Patients who have a limited understanding of the information but obey orders, although they have deep lethargy (they require painful stimuli to wake up), score 4.

• Patients who do not understand the information, or have problems understanding it, and who do not obey orders, score 5, regardless of the their GCS. Examples: unconscious patients, patients with intense dyspnoea or pain who do not understand the information, patients with severe cognitive impairment, agitated patients, who, in all the cases described, do not obey orders.

b.- Patient Communication

In this section, patient's ability to communicate with their environment is assessed:

• Patients who understand a common verbal language with their interlocutor and speak it, score 1.

• Patients who understand a common verbal language with their interlocutor but do not speak it, although they use some other alternative effective language to communicate, score 2. Example: dumb patients with dysarthria, patients with expressive aphasia who can communicate through an alternative method.

• Patients who do not understand a common verbal language with their interlocutor, who do not speak it, but who use some other alternative effective language to communicate, score 3. Example: foreign patients with whom we cannot communicate given that they do not speak our language, but who are able to communicate by means of an alternative method (in the previous case, if we spoke their language the score would be 1).

• Patients who understand a common verbal language with their interlocutor but do not speak it and who cannot use any other alternative effective language to communicate, score 4. Example: patient with stroke, conscious, who understands

verbal information but cannot communicate effectively through an alternative method.

• Patients who do not understand a common verbal language with their interlocutor and who cannot communicate through another type of effective alternative language, score 5. Example: patients with deterioration of the level of consciousness, with processes that impede the understanding of information (dementia, stroke), subject to deep sedation, etc.

Alternative communication is defined as the transmission of information through any common code to the interlocutors (signs, signals, writing, drawings ...).

c.- Risk factors

• Patients with no risk, score 1.

• Patients who present any mild senso-perceptive deterioration that allows them to live independently (for example, the use of glasses, hearing aids) and those who are overweight, score 2.

• Patients older than 70 years score 3.

• Patients who take more than 5 drugs and/or with chronic pain, score 4.

• Patients who have two or more of the above-mentioned factors in the previous score levels of 3 and 4, those with a history of falls, severe sensory-impaired impairment (blindness, profound deafness, muteness), with morbid obesity or with an inability to protect themselves (disoriented or confused patients, with cognitive deterioration, malnourished, unconscious, with significant impairment of mobility ...), score 5.

d.- Threats and patient coping for the maintenance of health

This section evaluates how the patient deals with their health situation, as well as the ability to protect against external threats.

• Patients who correctly perceive the seriousness of their process and their health situation, perceive the benefits of the therapeutic plan, do not identify barriers that limit their follow-up, and do not present anxiety or psychomotor agitation, score

1. In the text, it is expressed as SV ok, B ok, BA ok, AN ok (SV: Severity, B: Benefits, BA: Barriers, AN: Anxiety).

• Patients who present mild or moderate anxiety but maintain self-control and follow the prescribed therapeutic plan, score 2.

In the text, it is expressed as AN – or AN - -. Example: the common anxiety that any patient feels when suffering an acute process that threatens to his/her integrity.

• Patients who present minimization and/or denial of the severity of their process or perceive little or no benefit in following the prescribed therapeutic plan, or barriers to following it, score 3. All of these situations should be self-limited and solved without interventions by the care team. In the text, it is represented as SV-, B-, BA-.

Example: A patient who is also a family caregiver and refuses to be transferred to the hospital because he/she feels that the care of his/her relative could be jeopardized.

• Patients who present disabling anxiety, without agitation, minimization and/or denial of the severity of their process or perceive little or no benefit in following the prescribed therapeutic plan, or barriers to following it, score 4. All of these situations require the intervention of the emergency team. In the text, it is expressed as SV-, B-, BA-, AN- --

• Patients who present psychomotor agitation, who have carried out attempts or threats of violence against themselves, or those around them, or who are incapable of self-protection against external aggressions (disoriented or confused patients with cognitive impairment, malnourished, unconscious, with significant impairment of mobility ...), score 5.

MEDICAL INTERVENTIONS

• The absence of catheters or drainages, or if no drug is administrated, score 1.

• Patients who have inserted venous or intraosseous catheters, urethral catheters, as well as any type of drainage, score 2

- Patients who have been administered drugs with hypotensive therapeutic effect, with side effects of hypotension or dizziness, or that produce drowsiness, or are administered by an oral route (OR), intramuscular injection (II), sublingual route (SLR) or subcutaneous route (SCR), score 3.

- Patients who have been administered drugs with hypotensive therapeutic effect, with side effects of hypotension or dizziness, antiarrhythmic, antiepileptic, or opioids, via an intravenously (IV) or intraosseous (IO) route, score 4.

- Patients who have been administered drugs with sedative and/or muscle relaxant effect, via IV, IO, intrarectal (IR), or intranasal (IN) route, score 5.
